# Operative vs Non-Operative Management in Sterile Necrotizing Pancreatitis

**DOI:** 10.1155/1997/30279

**Published:** 1997

**Authors:** Edward L. Bradley

**Affiliations:** Department of Surgery State University of New York (Buffalo) Buffalo General Hospital 100 High Street Buffalo New York 14203 USA

## Abstract

*Background*: The clinical management of sterile pancreatic necrosis is still a matter of debate. In this study we analyzed the clinical course and outcome of patients with sterile necrotizing pancreatitis treated surgically versus nonsurgically.

*Study Design*: Between May 1982 and December 1993, 249 patients with necrotizing pancreatitis (NP) entered this study, of which 172 (69 percent) had intraoperatively or fine needle aspiration-proven sterile NP. One hundred seven of 172 patients underwent surgery (S group) with necrosectomy and continuous postoperative closed lavage and 65 of 172 were treated by nonsurgical means (NS group).

*Results*: Median Ranson and admission APACHE II scores were 4.7 (range, 1 to 10) and 11 (range, 1 to 29) in the S group, significantly higher than those in the NS group with 3.0 (range, 0 to 6) (*p*=0.022) and 8 (range, 1 to 23) (*p*=0.036). After 48 hours of intensive care treatment, APACHE II scores persisted at 10.5 (range, 1 to 29) in the S group and decreased to 6 (range, 0 to 15) (*p*=0.013) in the NS patients. Median Creactive protein (CRP) levels on admission were 179 mg/L and 68.5 mg/L (*p*=0.023), respectively. Within 72 hours, 61 (94 percent) of 65 NS-managed patients responded to intensive care therapy, whereas organ complications persisted or increased and thus led to surgery in the S group. Mortality rates were 13.1 percent in the surgically treated patients and 6.2 percent in the nonsurgically treated patients (*p*=NS).

*Conclusions*: Most patients with limited and sterile pancreatic necrosis respond to intensive care treatment. Indication for surgery in sterile NP should be based on persisting or advancing organ complications despite intensive care therapy. APACHE II scores and adraission CRP levels represent a helpful tool in decision making for surgical or nonsurgical management of NP.


In doubtful cases and in patients with high perioperative risk, a laparoscopy has been performed and in most cases the lesser sac was opened and inspected. With this procedure, we were able to decrease the number of unnecessary laparotomies significantly. In times of cost efficiency and limited resources new diagnostic measures have to be tested against non-invasive cheaper methods. As stated also by the authors, these new methods have to be evaluated against conventional investigations in prospective comparative studies.

### REFERENCES

1. Trede, M., Schwall, G. and Saeger, H.D. (1990) Survival after pancreatoduodenectomy: 118 consecutive resections without an operative mortality. *Ann Surg*; **211**, 447–458.
2. Rösch, T., Braig, C. and Gain, T. *et al.* (1992) Staging of pancreatic and ampullary carcinoma by endoscopic ultrasonography: comparison with conventional sonography, computed tomography, and angiography. *Gastroenterology*; **102**, 188–199.

Hans G Beger, MD
Professor of Surgery, FACS
Head of Department of Surgery
Michael H. Schoenberg MD
Associate Professor
University Hospital of Surgery
Steinhoevelstrasse 9
89075 Ulm
GERMANY

### OPERATIVE VS NON-OPERATIVE MANAGEMENT IN STERILE NECROTIZING PANCREATITIS

### ABSTRACT

*Rau, B., Pralle, U., Uhl, W., Schoenberg, M.H. and Beger, H.G. (1995) Management of sterile necrosis in instances of severe acute pancreatitis. Journal of The American College of Surgeons, 181: 279–288.*

***Background:*** **The clinical management of sterile pancreatic necrosis is still a matter of debate. In this study we analyzed the clinical course and outcome of patients with sterile necrotizing pancreatitis treated surgically versus nonsurgically.**
***Study Design:*** **Between May 1982 and December 1993, 249 patients with necrotizing pancreatitis (NP) entered this study, of which 172 (69 percent) had intraoperatively or fine needle aspiration-proven sterile NP. One hundred seven of 172 patients underwent surgery (S group) with necrosectomy and continuous postoperative closed lavage and 65 of 172 were treated by nonsurgical means (NS group).**
***Results:*** **Median Ranson and admission APACHE II scores were 4.7 (range, 1 to 10) and 11 (range, 1 to 29) in the S group, significantly higher than those in the NS group with 3.0 (range, 0 to 6) ($p$=0.022) and 8 (range, 1 to 23) ($p$=0.036). After 48 hours of intensive care treatment, APACHE II scores persisted at 10.5 (range, 1 to 29) in the S group and decreased to 6 (range, 0 to 15) ($p$=0.013) in the NS patients. Median C-reactive protein (CRP) levels on admission were 179 mg/L and 68.5 mg/L ($p$=0.023), respectively. Within 72 hours, 61 (94 percent) of 65 NS-managed patients responded to intensive care therapy, whereas organ complications persisted or increased and thus led to surgery in the S group. Mortality rates were 13.1 percent in the surgically treated patients and 6.2 percent in the nonsurgically treated patients ($p$=NS).**
***Conclusions:*** **Most patients with limited and sterile pancreatic necrosis respond to intensive care treatment. Indication for surgery in sterile NP should be based on**

**persisting or advancing organ complications despite intensive care therapy. APACHE II scores and admission CRP levels represent a helpful tool in decision making for surgical or nonsurgical management of NP. *J. Am. Coll. Surg.*, 1995, 181: 279–288.**

KEYWORDS: Necrotising pancreatitis pancreatitis acute pancreatic necrosectomy

### PAPER DISCUSSION

Indications for surgical intervention in patients with acute necrotizing pancreatitis are continuing to evolve. Despite almost universal acceptance of the necessity for surgical debridement in *infected* pancreatic necrosis, whether or not surgical necrosectomy offers any advantage to patients with *sterile* pancreatic necrosis has been problematic.

In this thoughtful and comprehensive retrospective analysis of a single institutional experience over an 11 year period, Dr. Rau and her colleagues have presented us with the indications for surgery in sterile pancreatic necrosis(SPN) as currently practiced in Ulm. Of 172 patients with SPN initially treated with intensive medical therapy, 107 were selected for surgery within 72 hours either because of "persisting organ failure" (72 patients), or "abdominal complications (for example; ileus, peritonitis, choledochal stenosis, etc.)" (32 patients). In keeping with developing knowledge, the 62% current surgical intervention rate from this center represents a downward modification from their original recommendation for global debridement of pancreatic necrosis[1]. The surgical approach consisted of necrosectomy and lesser sac lavage, performed in almost all cases within 7 days of the onset of illness. Sixty-five other patients with SPN who demonstrated improvement within the 72 hour period did not undergo surgery. Unfortunately, significant differences between the surgical and non-surgical groups in APACHE II scores (11 vs. 8), and in the extent of pancreatic necrosis (42 patients > 30% necrosis vs. 22 patients > 30% necrosis), precluded using the non-operative group as controls. Observed adverse increases in the surgical group with regard to mortality (13% vs. 6%), days of ICU treatment (16.5 vs. 7.0), days of hospitalization (44.5 vs. 20.0), and complications such as secondary abscess (29 vs. 1), and sepsis (45 vs. 5), were attributed by the authors to "...the higher severity of the disease...".

However, it is equally likely that the post-operative pancreatic infections which developed in 54% of the 72 surgical patients with SPN could have *caused* the excessive mortality and morbidity observed in the surgical group. Indeed, it is more than likely than not that the observed secondary pancreatic infections occurred as a *consequence* of exposing sterile injured tissues to ambient flora by the process of surgical debridment and prolonged drainage. In a collected group of 191 patients with SPN undergoing debridement (Table 1), 30% developed post-operative infections in the residual pancreatic tissue (2–4). Moreover, the high overall mortality rate of 18% (36/191) was made even worse by the realization that when surgically-induced infection did occur, the mortality rate was 63% (36/57). While we cannot know with absolute certainty that these post-operative pancreatic infections were surgically induced, infections are far less likely to occur in patients with comparable extent of necrosis treated without surgery (4–8). Other complications reported by Rau *et al.*, such as the development of intestinal fistulas in 27% of cases and the necessity for re-operation in 39% of patients, are directly attributable to surgical intervention and are less disputable.

Dismissing for the moment the possibility that surgical debridement of SPN is actually harmful, is there any persuasive evidence that operative intervention in these cases is beneficial? Since the tacit assumption underlying Professor Beger's surgical approach to SPN is that debridement will result in a reduction in mortality, it is reasonable to ask whether the surgical approach does in fact improve mortality risk in these patients. Moreover, because the major stated surgical indication in their series was persistent organ failure, is there any evidence that surgical debridement improves the course of organ insufficiency in patients with SPN? It is axiomatic that meaningful evaluation of any putative therapeutic approach requires comparison to an appropriate control

**Table 1** Risk of Iatrogenic Pancreatic Infection Following Surgical Debridement of Sterile Pancreatic Necrosis

| | *Number of Patients* | *Pancreatic Infections* | *Mortality due to Infection* |
|---|---|---|---|
| Smadja & Bismuth | 38 | 14(37%) | 9(24%) |
| Widdison *et al.** | 130 | 37(28%) | 22(17%) |
| Uomo *et al.* | 23 | 6(26%) | 5(22%) |
| Totals (avg) | 191 | 57(30%) | 36(19%) |

*Collected Series group. Since a population of unoperated patients with equally severe SPN was notably absent from their report, we are forced to turn to natural history information in order to evaluate their approach to management.

In collected series of 287 unoperated patients with SPN (4–8), directly comparable to the Ulm series in that the average extent of pancreatic necrosis was 54%, single or multiple organ failure existed in 39%, and equivalent indicators of severity were present, the overall mortality rate was found to be 9.7% (Table 2). When this mortality rate derived from these prospective studies is compared to the retrospective 13% surgical mortality reported by Rau *et al.*, no advantage to surgical intervention in comparable patients is apparent. Moreover, there is little available persuasive evidence that surgical intervention favorably affects either the incidence or the course of organ failure. Smadja and Bismuth were unable to detect any beneficial effects of necrosectomy on pre-existing organ failure in thier patients, and in fact concluded that surgical intervention exacerbated organ insufficiency[2]. Similar negative conclusions regarding surgical amelioration of organ failure have been reached by other workers[3,4]. From a theoretic standpoint, if organ failure were due to the remote effects of circulating noxious substances released by pancreatic necrosis, then removal of the necrotic tissues should result in a relatively rapid reversal of organ dysfunction, given the reasonable supposition that the half-lives of these serum substances should be short. Yet many studies have failed to demonstrate any conclusive benefit to organ failure following with debridement of SPN. Since continuously elevated APACHE II scores usually reflect persisting organ failure, and the average post operative APACHE II scores in the Ulm patients remained relatively constant for at least 7 days following surgery, it is even difficult to demonstrate any favorable organ effects to debridement in *their* cases.

If there is no unequivocal survival advantage to surgery in comparable patients, no demonstrable amelioration in existing organ failure, and if the potential exists for actual harm in the form of intestinal fistula formation and surgically induced infection, it is not clear that the case has been made for programmatic surgical debridement in patients with SPN.

Even though the overwhelming majority of patients with SPN do not appear to benefit from necrosectomy, it remains possible that smaller sub-groups of patients might gain from surgical intervention. In our experience, the only patients for whom debridement of SPN is of unquestioned value are those who develop abdominal pain and hyperamylasemia resulting from attempts at oral feeding after 4–6 weeks of non-operative therapy. This clinical configuration occurs in 5–7% of cases, and is usually due to necrosis-induced changes in the pancreatic ductal system. Accordingly, the principal issue of contention in this debate is not whether surgical debridement should be done in *any* patient with SPN, but rather how frequently it is necessary.

It would seem that additional validation is required before we can accept the Ulm proposals that the *majority* of patients with SPN require operative intervention, or that the necessity for surgery can be predicted by the duration of organ failure, specific serum levels of C-reactive protein, or APACHE II thresholds. Moreover, the increasing amount of natural history data generated from comparable non-operated patients, has placed the burden of proof placed squarely upon the shoulders of those advocating surgery. It is becoming increasingly clear to many that in the near future surgical debridement of sterile necrotizing pancreatitis will become the exception rather than the rule.

**Table 2** Non-Operative Management of Sterile Pancreatic Necrosis

| | *Year* | *Patients (N)* | *Extent of Necrosis (AVG%)* | *Organ Failure (N)* | *Severity (AVG#)* | *Mortality (%)* |
|---|---|---|---|---|---|---|
| Bradley et al. | 1991 & FF* | 40 | 58 | 19 | 14A | 10.0 |
| Guillames *et al.* | 1992* | 27 | NS | NS | NS | 7.4 |
| Andersson *et al.* | 1994 | 59 | NS | NS | NS | 10.0 |
| Uhl *et al.* | 1995* | 15 | 35 | 10 | 7A | 0 |
| Uomo *et al.* | 1996* | 146 | 55 | 49 | >3R(89%) | 9.5 |
| Total | | 287 | 54% | 39% | – | 9.7% |

* = Prospective
NS = Not Stated
A = Apache II Points
R = Ranson Signs

### BIBLIOGRAPHY

1. Beger, H.G., Krautzberger, W. and Bittner, R. *et al.* (1985) Results of surgical treatment of necrotizing pancreatitis. *World J Surg.*, **6**, 972–979.
2. Smadja, C. and Bismuth, H.: (1986) Pancreatic debridement in acute necrotizing pancreatitis: An obsolete procedure. *Brit J Surg.*, **73**, 408–410.
3. Widdison, A.L., Alvarez, C. and Reber, H.A.: (1991) Surgical intervention in acute pancreatitis: When and how. *Pancreas.*, **6**, 544–551.
4. Uomo, G., Visconti, M. and Manes, G. *et al*: (1996) Nonsurgical treatment of acute necrotizing pancreatitis. *Pancreas.*, **12**, 142–148.
5. Bradley EL III. Debridement is rarely necessary in patients with sterile pancreatic necrosis. *Pancreas* 1996 (in press).
6. Gillaumes, S., Blanco, I., Clave, P. and Marruecos, L: (1992) Non-operative management of necrosis in acute pancreatitis. *Pancreas.*, 7, 740.
7. Andersson, R., Steinbach, R., Leveau, P. Ihse I (1994) Results of primarily non-operative treatment in severe acute pancreatitis. Abstracts of the International Hepatobiliary Pancreatic Association, p.91.
8. Uhl, W., Stupnicki, A., Schmid, S. T., Vogel, R. and Büchler, M.W. The role of surgery in severe acute pancreatitis: Lessons learned from the past. Presented at the Pancreas Club, San Diego, May 14, 1995.

Edward L Bradley, III, MD
Department of Surgery
State University of New York (Buffalo)
Buffalo General Hospital
100 High Street
Buffalo, New York 14203
United States of America

### JEJUNUM OR STOMACH FOR THE PANCREATIC ANASTOMOSIS AFTER PANCREATICODUODENECTOMY

### ABSTRACT

*Yeo, C.J., Cameron, J.L., Maher, M.M., Sauter, P.K., Zahurak, M.L., Talamini, M.A., Lillemoe, K.D. and Pitt, H.A. (1995) A prospective randomized trial of pancreaticogastrostomy versus pancreaticojejunostomy after pancreaticoduodenectomy. Annals of Surgery; 222: 580–592.*

***Objective:*** **The authors hypothesized that pancreaticogastrostomy is safer than pancreaticojejunostomy after pancreaticoduodenectomy and less likely to be associated with a postoperative pancreatic fistula.**
***Summary Background Data:*** **Pancreatic fistula is a leading cause of morbidity and mortality after pancreaticoduodenectomy, occurring in 10% to 20% of patients. Nonrandomized reports have suggested that pancreaticogastrostomy is less likely than pancreaticojejunostomy to be associated with postoperative complications.**
***Methods:*** **Between May 1993 and January 1995, the findings for 145 patients were analyzed in this prospective trial at The Johns Hopkins Hospital. After giving their appropriate preoperative informed consent, patients were randomly assigned to pancreaticogastrostomy or pancreaticojejunostomy after completion of the pancreaticoduodenal resection. All pancreatic anastomoses were performed in two layers without pancreatic duct stents and with closed suction drainage. Pancreatic fistula was defined as drainage of greater than 50 mL of amylase-rich fluid on or after postoperative day 10.**
***Results:*** **The pancreaticogastrostomy ($n$=73) and pancreaticojejunostomy ($n$=72) groups were comparable with regard to multiple parameters, including demographics, medical history, preoperative laboratory values, and intraoperative factors, such as operative time, blood transfusions, pancreatic texture, length of pancreatic remnant mobilized, and pancreatic duct diameter. The overall incidence of pancreatic fistula after pancreaticoduodenectomy was 11.7% (17/145). The incidence of pancreatic fistula was similar for the pancreaticogastrostomy (12.3%) and pancreaticojejunostomy (11.1%) groups. Pancreatisc fistula was associated with a significant prolongation of postoperative hospital stay (36±5 vs. 15±1 days) ($p$<0.001). Factors significantly increasing the risk of pancreatic fistula by univariate logistic regression analysis**

